# The accuracy of international and national fetal growth charts in detecting small-for-gestational-age infants using the Lambda-Mu-Sigma method

**DOI:** 10.3389/fsurg.2023.1123948

**Published:** 2023-04-11

**Authors:** Shier Nee Saw, Mei Cee Lim, Chuan Nyen Liew, Azanna Ahmad Kamar, Sofiah Sulaiman, Rahmah Saaid, Chu Kiong Loo

**Affiliations:** ^1^ Department of Artificial Intelligence, Faculty of Computer Science and Information Technology, Universiti Malaya, Kuala Lumpur, Malaysia; ^2^ Department of Paediatrics, Faculty of Medicine, Universiti Malaya, Kuala Lumpur, Malaysia; ^3^ Department of Obstetrics and Gynaecology, Faculty of Medicine, Universiti Malaya, Kuala Lumpur, Malaysia

**Keywords:** small-for-gestational-age, estimated fetal weight, growth chart, reference chart, fetal growth, INTERGROWTH-21st growth chart, Hadlock growth chart, WHO growth charts

## Abstract

**Objective:**

To construct a national fetal growth chart using retrospective data and compared its diagnostic accuracy in predicting SGA at birth with existing international growth charts.

**Method:**

This is a retrospective study where datasets from May 2011 to Apr 2020 were extracted to construct the fetal growth chart using the Lambda-Mu-Sigma method. SGA is defined as birth weight <10th centile. The local growth chart's diagnostic accuracy in detecting SGA at birth was evaluated using datasets from May 2020 to Apr 2021 and was compared with the WHO, Hadlock, and INTERGROWTH-21st charts. Balanced accuracy, sensitivity, and specificity were reported.

**Results:**

A total of 68,897 scans were collected and five biometric growth charts were constructed. Our national growth chart achieved an accuracy of 69% and a sensitivity of 42% in identifying SGA at birth. The WHO chart showed similar diagnostic performance as our national growth chart, followed by the Hadlock (67% accuracy and 38% sensitivity) and INTERGROWTH-21st (57% accuracy and 19% sensitivity). The specificities for all charts were 95–96%. All growth charts showed higher accuracy in the third trimester, with an improvement of 8–16%, as compared to that in the second trimester.

**Conclusion:**

Using the Hadlock and INTERGROWTH-21st chart in the Malaysian population may results in misdiagnose of SGA. Our population local chart has slightly higher accuracy in predicting preterm SGA in the second trimester which can enable earlier intervention for babies who are detected as SGA. All growth charts' diagnostic accuracies were poor in the second trimester, suggesting the need of improvising alternative techniques for early detection of SGA to improve fetus outcomes.

## Introduction

Small-for-gestational-age (SGA) refers to newborns with birth weights less than the 10th centile who may have a higher risk of adverse perinatal and long-term health outcomes due to fetal growth restriction (FGR) (1, 2). FGR refers to a fetus that fails to reach its genetically determined growth due to multiple factors, including maternal conditions, placental insufficiency, or fetal-related causes. FGR is the main risk factor for stillbirth and the stillbirth rate (per 1,000 birth) increased from 4.2 to 9.2 if FGR remains undetected before delivery (3). FGR detection before birth is essential as the risk of adverse outcomes can reduce four-fold if proper antenatal care is given (4).

Current clinical standards in detecting SGA include fetal growth assessment *via* routine ultrasonography where fetal weight is compared with a population growth chart. The Hadlock chart is commonly accepted worldwide (5). Various fetal reference growth charts have been proposed by the INTERGROWTH-21st project (6), the NICHD Fetal Growth Study (7), and the World Health Organization (WHO) (8). Discussion on which growth charts should be adopted in the local cohort is ongoing because the choice of growth chart has profound implications on the clinical management of fetal growth assessment (9, 10).

Malaysia has 440 K live birth per year and 50 stillbirths rate per 10 K birth (11). The stillbirth rate due to FGR is 16.5 per 1,000 birth (12). The motivation to construct a national fetal growth chart is due to the increased stillbirth rate in Malaysia, leading to the failure to achieve the UN's' Millennium Development Goals (MDGs) of child mortality reduction and improvement of maternal health. Evidence has shown that fetal weight is greatly influenced by genetic and demographic factors (13–16). To date, Malaysia lacks a national fetal growth chart and is using international growth charts, which are created based on the Caucasian population (5), in fetal growth assessment which may underdiagnose SGA.

The first objective of this study is to investigate the diagnostic performance of various international growth charts in predicting SGA at birth. The second objective is to construct a national fetal growth chart using ten-year retrospective local data and compare its diagnostic performance in predicting SGA at birth with existing international growth charts.

## Methods

### Subjects

The study protocol was approved by the University of Malaya Medical Center, Medical Research Ethical Committee (MREC) with MECID.No: 2021329-9997. All the data involved in the current research project originated from Pusat Perubatan Universiti Malaya (PPUM), a government-funded medical institution in Kuala Lumpur, Malaysia. Prenatal data from May 2011 to Apr 2021 were extracted from the system. Scan records with missing values, pregnancy with multiparity, stillbirth, and consist of values that fall outside the range of three times the interquartile range were removed. Only one ultrasound measurement was used for each fetus. We have a total of 68,897 scans from May 2011 to Apr 2021. A total of seven features were extracted from the scan records and are described in Table 1. All ultrasound measurements were performed by sonographers certified by the Fetal Medicine Foundation.

**Table 1:** Description of features collected in prenatal data.

Feature	Description
Pregnancy ID	Identity code of the patient.
Gestational Age	Dating by last menstrual period or crump-lump length.
Biparietal Diameter (BPD)	A measurement of the diameter of the fetus's skull, measured on the axis plane of the fetus vertex, from one parietal bone to the other.
Head Circumference (HC)	A measurement of the circumference of the fetus's skull, measured on the axis plane of the fetus vertex, head-around of the fetus's skull.
Abdominal Circumference (AC)	A measurement of the circumference of the fetus's abdomen, measured on the transverse section through the upper abdomen.
Femur Length (FL)	A measurement of the long bone in the fetus's thigh, measured from the blunt end of the bone to the shaft.
Estimated Fetal Weight (EFW)	An estimation of the weight of the fetus based on ultrasonographic measurement using the Hadlock formula.

### Outcome

Our primary outcome was to predict SGA at birth. We defined SGA at birth when the birth weight is less than the 10th centile, based on the INTERGROWTH-21st preterm and term birth weight chart (17, 18). If birth weight is above the 10th centile, it is defined as appropriate gestational age (AGA) at birth.

### Development of fetal growth reference curves

We adopted the first nine-year datasets from May 2011 to Apr 2020 (*n* = 67,063 scans) to generate the fetal growth chart using the Lambda-Mu-Sigma (LMS) statistical method (19). The LMS method is an established method in creating reference charts (20–22). The LMS method summarizes the distribution of fetal biometrics by gestational age in three aspects, which are Lambda (L) which indicates the skewness of the distribution of fetal biometrics by Box-Cox transformation power, Mu (M) which indicates the Median of the fetal biometric, and lastly the Sigma (S) that indicates the coefficient of the variation of the fetal biometric. Nature smoothing spline function was applied to obtain the smoothed value of Lambda, Mu, and Sigma for each gestational age, these values were then fed into the equation as followed to calculate the percentile value in a particular gestational age:

(1) $$C\;=\;M\;(1+(L\cdot S\cdot Z){)}^{\frac{1}{L}}$$

where *C* is the unit value at a particular percentile level to be calculated; *M, L*, and *S* are the Mu (median), Lambda (skewness of distribution), and Sigma (coefficient of variation) as described previously; *Z* is the corresponding Z-Score of the percentile in a normalized distribution (e.g. for the value of percentile 2.5th, 5th, 10th, 25th, 50th, 75th, 90th, 95th, and 97.5th, *Z* will be substituted as −1.960, −1.881, −1.645, −1.282, −0.675, 0, 0.675, and 1.282, 1.645, 1.881, 1.960). The generated unit value at a particular percentile using the LMS method was aggregated and presented as a fetal growth reference curved with intervals of one week by gestational age.

The difference between our LMS fetal growth chart and the growth chart from WHO (8), INTERGROWTH-21st (6, 23), and Hadlock (5) was compared using relative percentage difference (Equation 2).

(2) $$\begin{array}{rl} & \mathrm{Relative}\;\text{\%}\;\mathrm{Difference}\;=\\ & \left(\frac{\begin{array}{c} \mathrm{International}\;\mathrm{Growth}\;\mathrm{Chart}\;\mathrm{Centile}\;\mathrm{Value}\\ \;-\;\mathrm{LMS}\;\mathrm{Centile}\;\mathrm{Value} \end{array}}{\mathrm{LMS}\;\mathrm{Centile}}\;\right)\times 100\text{\%} \end{array}$$

### Performance analysis of fetal growth reference charts in predicting SGA at birth

The 10th-year datasets (May 2020 to Apr 2021, *n* = 1,834 scans) were used to evaluate the accuracy of our fetal growth chart in predicting SGA at birth. Fetuses with EFW that fall below the 10th centile were predicted as SGA at birth while fetuses with EFWs above the 10th centile were predicted as AGA at birth.

The evaluation was divided into three parts. First, the performance of the local growth chart, generated using local data with the LMS method, in predicting SGA at birth was evaluated. Second, we analyzed the performance of the local growth chart in predicting SGA at birth in preterm and term infants. A preterm infant is defined as when an infant is born before the 37th week while a term infant is defined as when an infant is born after the 37th week (24). Third, we further analyze the performance of the chart in predicting SGA at birth using second and third-trimester data for term and preterm infants. Any data with gestational age between the 13th week and 27th week was identified as “second trimester” and data with gestational age more than or equal to 27th was identified as “third trimester”.

The performance of the local growth chart and WHO (8), INTERGROWTH-21st (6, 23), and Hadlock (5) fetal growth charts in predicting SGA at birth were compared. The dataset is imbalanced as the number of SGA is much lesser than the number of AGA cases, balanced accuracy is used instead of accuracy (25). Balanced accuracy is the mean of sensitivity and specificity. Balanced accuracy, sensitivity, and specificity were reported.

### Statistical analysis

Statistical analysis was performed to test if there were any significant differences between the AGA and SGA. For continuous variables, normality test was performed to check for data distribution. If data is normally distributed, Student T-test is used else non-parametric Mann-Whitney test is used for analysis. For categorical variables, chi-square test was performed to determine if there is a significant difference between AGA and SGA. The data were deemed significantly different if *p* < 0.05.

## Results

### Patients characteristics

**Figure 1:**
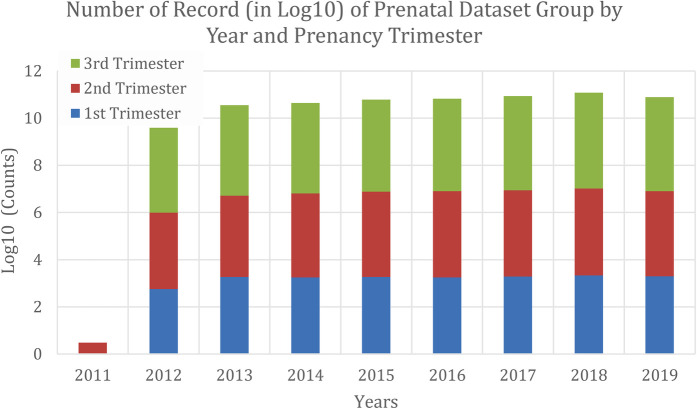
Data distribution from Apr 2011 to May 2020 that was used to generate local fetal growth chart curves.

Figure 1 shows the data distribution used to generate our fetal growth chart curves. In 2011, UMMC had just started using a proper electronic system for keeping patient records and thus the datasets in 2011 were limited and all of them were in 2nd trimester. Table 2 tabulates the patients' characteristics from May 2020 to Apr 2021, which was used for evaluating the accuracy of various growth charts in identifying SGA at birth. There are 781 newborns with 706 AGA and 75 SGA, defined using the birth weight of the infants. There were significant differences between AGA and SGA fetuses, where the occurrence of maternal hypertension, pre-eclampsia, gender, as well as the birth anthropometric measurements of birth weight, length, and head circumference differ. No significant differences were observed for maternal age, anemia, gestational age at birth, and APGAR score for 1 min and 5 min.

**Table 2:** Patient characteristics from May 2020–Apr 2021.

*N* = 781	AGA (*N* = 706)	SGA (*N* = 75)	*p*-value
Mother Age	34.16 ± 3.96	34.63 ± 3.88	0.301
Anemia	28 (4.0%)	6 (8.0%)	0.104
Hypertension*	58 (8.2%)	14 (18.6%)	0.003
Pre-eclampsia*	-	2 (2.7%)	<0.0001
**Gestational Age at birth (week)**
Term	38.09 ± 0.91	38.10 ± 0.99	0.981
Preterm	35.64 ± 0.77	35.24 ± 1.09	0.105
**Gender** *
Female	334 (47.3%)	46 (61.3%)	0.021
Male	372 (52.7%)	29 (38.7%)	
Birth weight (g)*	3091.86 ± 387.06	2341.93 ± 339.60	<0.0001
Birth length (mm)*	47.70 ± 2.13	45.02 ± 2.37	<0.0001
Head circumference at birth (mm)*	33.65 ± 1.84	31.75 ± 1.32	<0.0001
APGAR score (1 min)	8.81 ± 0.90	8.79 ± 1.15	0.552
APGAR score (5 min)	9.90 ± 0.70	9.85 ± 1.16	0.519

Values shown for continuous variables are mean and standard deviation while categorical variables are counts (percentage) *N*: Number of newborn.

* indicates *p* < 0.05.

### Fetal growth reference curves

**Figure 2:**
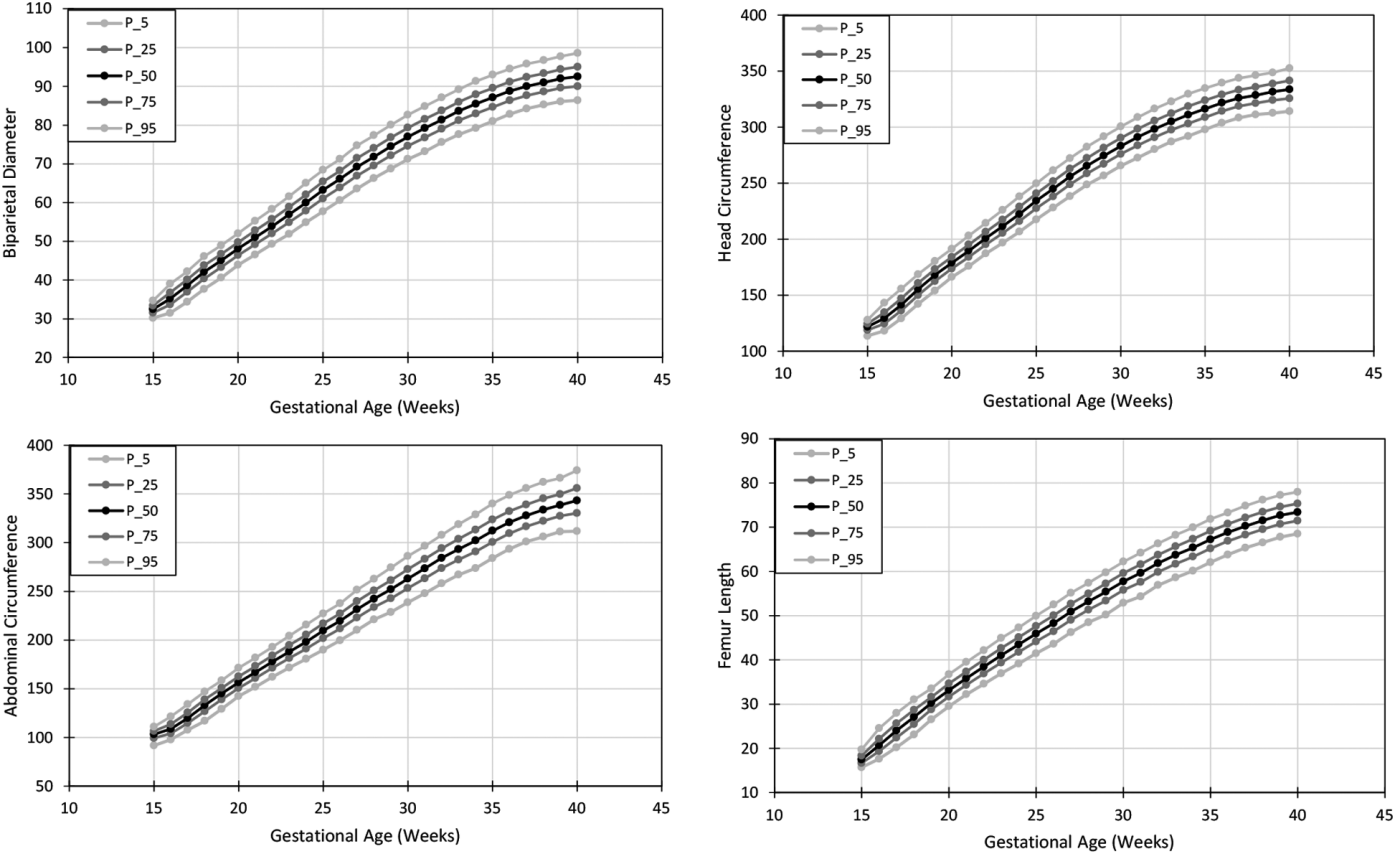
Local fetal growth reference charts for biparietal diameter, head circumference, abdominal circumference, and femur length using LMS method.

**Figure 3:**
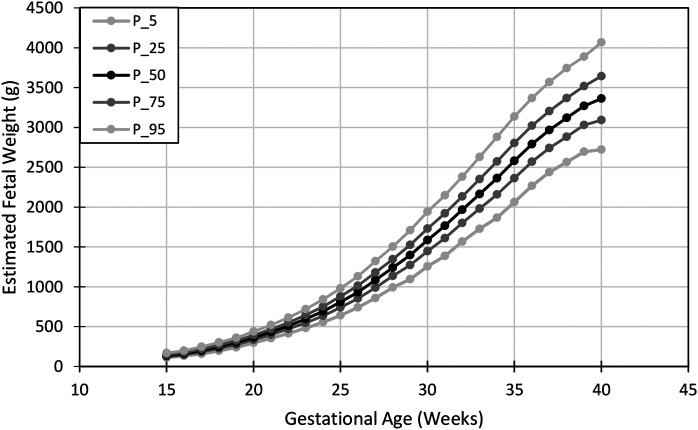
Local fetal growth reference curve for estimated fetal weight using LMS method.

Figures 2, 3 shows the reference charts for biparietal diameter, head circumference, abdominal circumference, femur length, and estimated fetal weight generated using the LMS method. Supplementary Tables S1–S5 show the centile estimations for completed weeks of gestation.

### Fetal growth reference curves comparison

**Figure 4:**
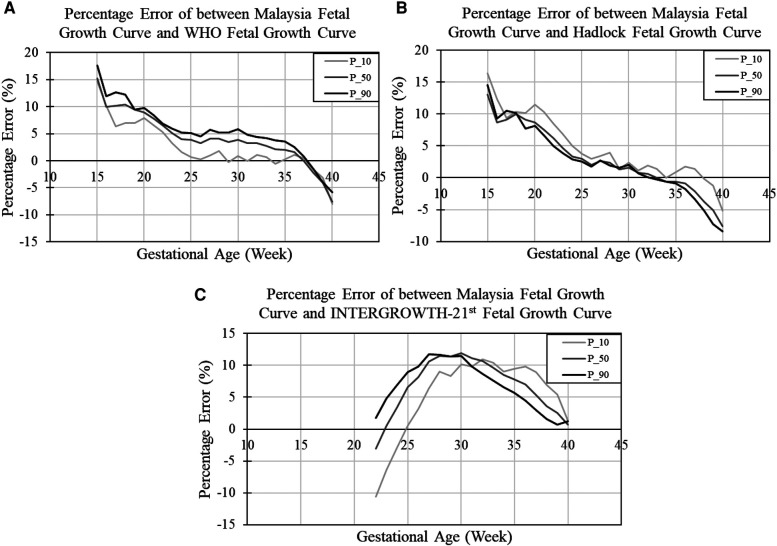
Relative percentage difference of estimated fetal weight between Malaysia and (**A**) WHO, (**B**) Hadlock, and (**C**) INTERGROWTH-21st fetal growth curves. *P*_10, *P*_50, and *P*_90 refer to percentage error when comparing the 10th centile, 50th centile, and 90th centile of the two growth charts, respectively.

Figure 4 shows the relative percentage difference of the EFW centile curve between the local generated curve and the WHO, INTERGROWH-21st^,^ and Hadlock centile curves. A positive percentage error indicates the percentile value of growth reference (WHO/ INTERGROWH-21st/Hadlock) is larger than the percentile value of growth reference developed in our study. In other words, the positive and negative percentage error representing fetal growth are over- and under-estimated, respectively, if international growth references (WHO/ INTERGROWH-21st/Hadlock) are adopted for fetal growth assessment in the local population.

In our study, we found that the discrepancy between our EFW chart and the WHO growth chart ranged from +18% to −10% across gestation, indicating an 18% of overestimation at early gestation and a 10% underestimation of fetus growth at late gestation. From Figure 4A, the WHO growth chart overestimated the FGR fetus growth (light grey line) and the overestimation of FGR fetus growth was 15% in the 15th week and dropped gradually to below 5% after the 23rd week. The percentage error for evaluating EFW below the 10th centile between our growth chart and WHO curve was small, approximately 0.56%, between 25th to 37th week.

The Hadlock curve exhibited a similar reducing trend of percentage error when compared to our local EFW curves. The percentage error was 16% in the 15th week and dropped to below 5^%^ after the 25th week. The percentage error for evaluating EFW below the 10th centile from 25th to the 37th week between our growth chart and Hadlock curve was approximately 2.03%.

As there were no information available before the 21st week in the INTEGROWTH-21st chart, the comparisons were only made between the 22nd to 40th week. From Figure 4C, the INTEGROWTH-21st chart exhibited a negative discrepancy between the 22nd to 25th week, indicating that some of the fetuses may be misdiagnosed as AGA. The discrepancy increased to positive after the 25th week and declined around the 32nd week.

### Evaluation of fetal growth reference chart in general

Table 3 shows the results of each fetal growth reference chart in predicting SGA at birth. Based on the result, we noticed that our fetal growth reference chart generated using the LMS method achieved similar balanced accuracy (69%), as the WHO chart. The recall for both local and WHO charts were similar, achieving 42% and 43%, respectively. The INTERGROWTH-21st chart had the poorest performance with a balanced accuracy of 57% and 19% recall. All charts had similar specificity of 95%–96%.

**Table 3:** Result of different fetal growth reference charts in predicting SGA at birth.

*n* = 1,834	Balanced Accuracy	Recall / Sensitivity	Specificity
Hadlock	67%	38%	96%
IG-21st	57%	19%	96%
*LMS (Ours)*	69%	42%	95%
WHO	69%	43%	96%

*n*, number of scans; IG-21st, INTERGROWTH-21st.

To further understand the performance of each chart in predicting SGA, we performed another analysis by segregating SGA into preterm and term SGA (Table 4). Based on the result, we observed a similar pattern as observed in Table 3, where the WHO and our local chart achieved the highest accuracy in predicting SGA for both preterm and term infants, followed by the Hadlock and INTERGROWTH-21st charts. For preterm infants, *via* the LMS method, the WHO and local growth chart achieved balanced accuracy of 76% and recall of 65% in predicting SGA at birth. The Hadlock chart had slightly lower accuracy and recall as compared to the WHO and local charts. The INTERGROWTH-21st chart showed the lowest balanced accuracy of 63% and recall of 31% for preterm SGA. For term infants, the WHO chart depicted the highest balanced accuracy, with 66% and recall of 35% for SGA, followed by our local growth chart with 65% balanced accuracy and 34% recall. The INTERGROWTH-21st chart, again, showed the lowest accuracy of 55% and recall of 14%. For preterm SGA, the INTERGROWTH-21st chart showed the highest specificity of 94%, followed by the Hadlock chart, with 90% specificity. The WHO and our local charts had slightly lower specificities, 88%. For the term SGA, all four charts showed similar specificity of approximately 96%.

**Table 4:** Result of different fetal growth reference charts in predicting SGA for preterm and term infants.

	Type of Growth Charts	Balanced Accuracy	Recall / Sensitivity	Specificity
Preterm (*n* = 228)	Hadlock	74%	59%	90%
	IG-21st	63%	31%	94%
	*LMS (Ours)*	*76%*	*65%*	*88%*
	WHO	76%	65%	88%
Term (*n* = 1606)	Hadlock	63%	30%	97%
	IG-21st	55%	14%	96%
	*LMS (Ours)*	*65%*	*34%*	*96%*
	WHO	66%	35%	96%

*n*: number of scans.

Table 5 shows the results of various fetal growth charts in predicting term and preterm SGA in the 2nd and 3rd trimesters. Interestingly, although the INTERGROWTH-21st chart had the poorest performance in Tables 3, 4, it had the highest balanced accuracy when predicting SGA in 2nd trimester but dropped greatly in 3rd trimester (Balanced Accuracy: Preterm SGA: 75% drop to 59% and Term SGA: 64% drop to 55%). The WHO chart had poor performance in 2nd trimester but improved substantially in 3rd trimester (Balanced Accuracy: Preterm SGA: 66% to 79% and Term SGA: 56% to 67%). The Hadlock chart achieved an average balanced accuracy of 52%–76%.

**Table 5:** Result of different fetal growth reference charts in predicting SGA for preterm and term infants in 2nd trimester and 3rd trimester.

	Type of Growth Charts	Balanced Accuracy	Recall / Sensitivity	Specificity
**Preterm SGA**
2nd Trimester (*n* = 49)	Hadlock	68%	36%	100%
	IG-21	75%	64%	87%
	*LMS (Ours)*	***70%***	***45%***	***95%***
	WHO	66%	36%	95%
3rd Trimester (*n* = 179)	Hadlock	76%	65%	87%
	IG-21	59%	23%	96%
	*LMS (Ours)*	***78%***	***70%***	***86%***
	WHO	79%	72%	86%
**Term SGA**
2nd Trimester (*n* = 310)	Hadlock	52%	6%	98%
	IG-21	64%	44%	83%
	*LMS (Ours)*	***58%***	***22%***	***95%***
	WHO	56%	17%	96%
3rd Trimester (*n* = 1296)	Hadlock	65%	33%	97%
	IG-21	55%	10%	99%
	*LMS (Ours)*	***66%***	***36%***	***97%***
	WHO	67%	37%	97%

*n*, number of scans.

As compared to the WHO and INTERGROWTH-21st charts, our local growth chart showed a more consistent trend in the 2nd and 3rd trimesters in predicting SGA at birth. The discrepancy of balanced accuracies between the 2nd and 3rd trimesters was as large as compared to the WHO and INTERGROWTH-21st charts. From Table 5 for preterm SGA, our local growth chart achieved 70% balanced accuracy in the 2nd trimester and increased to 78% in the 3rd trimester while for term SGA, our local growth chart shows 58% balanced accuracy in the 2nd trimester and improved to 66% in the 3rd trimester.

## Discussion

It is known that adopting an international fetal growth chart may not be suitable for certain population. For example, an Italian study and a population-based study in 15 European countries reported that using international growth charts results in underdiagnosed SGA and FGR fetuses being misclassified as normal growth respectively (9, 10). Furthermore, Asian population from specific areas such South East Asia, are relatively smaller in overall size compared to the white or Caucasian population (7), and thus, adopting an international growth chart in the Malaysian population may misdiagnose SGA or FGR.

Our study's major strength is the inclusion of a very large sample of live births over a span of nine years to construct a national fetal growth chart that can be used as a reference to Malaysia's population. We tested the performance of the local fetal growth chart in predicting SGA at birth using another independent dataset—the 10th-year data. The accuracy of our fetal growth chart in predicting SGA at birth was 69%, depicting similar diagnostic accuracy as the WHO chart, which was constructed with approximately 20% Asian population. Another important point from our study is that our local chart has higher accuracy and sensitivity in predicting preterm SGA at birth in the second trimester would allow possible interventions such as maternal supplementations (26, 27).

The Hadlock growth chart has been widely accepted and used in clinics for fetal growth assessment globally, including in Malaysia. However, we observed that the Hadlock growth chart did not show the best diagnostic accuracy in predicting SGA at birth, achieving only 38% sensitivity and 96% specificity (Table 3). When we analyzed the results in the second and third trimesters independently, the sensitivity of the Hadlock growth chart only increased to 49% in the third trimester, which was lower than other studies that reported a sensitivity of 62%–69% (28, 29). The NICHD study reported that the white population had significantly higher fetal growth as compared to the Asian (7), suggesting that adopting a Hadlock growth chart (Caucasian) in our cohort may underdiagnose SGA and hence results in low sensitivity. The same observation was also observed in Papua New Guinea, where the Hadlock chart overestimated the percentage of fetuses with EFW <10th centile (30). This result suggests that adopting the Hadlock chart in Malaysia healthcare institutions may require reconsideration.

Other studies reported that the INTERGROWTH-21st chart did not perform well in identifying SGA at birth (31, 32). For example, a substantial number of fetuses in the Chinese population were being misdiagnosed as at risk of small fetus size (high false positive) (31). In our study, we observed an opposite trend with a significant number of fetuses being misdiagnosis for normal size (high false negative). In fact, the INTERGROWTH-21st chart was the poorest in identifying SGA at birth among all the fetal growth charts. We reckon that the inadequate performance of the INTERGROWTH-21st chart could be due to the discrepancy in the population recruitment criteria where pregnancies with antenatal complications were excluded. The second reason could be EFW is computed using another formula, instead of the Hadlock formula, in the INTERGROWTH-21st study which may result in a discrepancy in EFW estimation (6, 23).

A past study reported that the degree of discrepancy between ultrasound EFW and birth weight increased with the number of days scans completed before delivery (33). This could explain the reason why we observed that all growth charts generally have lower diagnostic accuracy in the second trimester as compared to that in the third trimester, with an 8%–16% decline in performance (Table 5). Similar findings were also reported where the sensitivity of predicting SGA at birth in the second trimester was approximately 45% (34, 35). Fetal growth is a dynamic process where it can be affected by various factors such as maternal diet. As such, it is not surprising that the detection rate in the second trimester is poorer than in the third trimester.

Compared with the WHO growth chart, one advantage provided by our local population chart is that it is better at predicting preterm SGA in second trimester (Table 5). Preterm SGA is reported to have 13 times higher risk associated with mortality (Kc et al., 2015; García-Basteiro et al., 2017). An earlier recognition of SGA can improve neonatal prognosis and provide an earlier indication of placental disease (36). Detection of SGA in mid-pregnancy could imply that the mothers have a high level of stress, anxiety or depression (37). This information could be helpful to prenatal care planner in designing intervention program in reducing the risk of delivering SGA infant.

One of the study limitations is that we only consider EFW for SGA prediction and did not consider other important covariates, such as maternal variables. However, although customized fetal growth charts have been proposed to improve SGA detection, their predictive ability has been questioned due to methodology bias (38, 39). The second limitation is that the populations selected for this study were urban in Malaysia and thus applying our national growth chart in rural areas may require validation of the performance.

In conclusion, we have constructed five biometric growth charts: biparietal diameter, abdominal circumference, head circumference, femur length and estimated fetal weight. Our national growth chart achieved 69% accuracy in identifying SGA at birth. The WHO chart better reflects our local population compared to the Hadlock and INTERGROWTH-21st charts. Our population local chart has slightly higher accuracy in predicting preterm SGA in the second trimester which enable prompt identification to implement intervention to increase survival of these infants.

## Data Availability

The raw data supporting the conclusions of this article will be made available by the authors, without undue reservation.
