# Supplementary material for: The accuracy of international and national fetal growth charts in detecting small-for-gestational-age infants using the Lambda-Mu-Sigma method
Source: Front Surg. 2023 Apr 11;10:1123948. doi: 10.3389/fsurg.2023.1123948 (PMC10126230; doi:10.3389/fsurg.2023.1123948)
Supplement: Supplementary file 1 [file Datasheet1.pdf]

## APPENDIX

***Table 1 LMS-Generated Fetal Growth Reference (Biparietal Diameter)***

| GA | P_2.5 | P_3 | P_5 | P_10 | P_25 | P_50 | P_75 | P_90 | P_95 | P_97 | P_97.5 |
|---|---|---|---|---|---|---|---|---|---|---|---|
| **15** | 29.80 | 29.91 | 30.22 | 30.71 | 31.54 | 32.45 | 33.37 | 34.19 | 34.69 | 35.01 | 35.12 |
| **16** | 30.82 | 30.99 | 31.51 | 32.32 | 33.67 | 35.20 | 36.75 | 38.15 | 39.00 | 39.56 | 39.74 |
| **17** | 33.48 | 33.71 | 34.38 | 35.38 | 36.96 | 38.60 | 40.15 | 41.47 | 42.23 | 42.72 | 42.88 |
| **18** | 36.81 | 37.03 | 37.70 | 38.71 | 40.34 | 42.09 | 43.78 | 45.25 | 46.11 | 46.66 | 46.84 |
| **19** | 39.65 | 39.89 | 40.59 | 41.64 | 43.31 | 45.06 | 46.72 | 48.14 | 48.96 | 49.48 | 49.66 |
| **20** | 43.07 | 43.28 | 43.91 | 44.86 | 46.41 | 48.09 | 49.73 | 51.16 | 52.01 | 52.55 | 52.73 |
| **21** | 45.68 | 45.90 | 46.56 | 47.56 | 49.21 | 51.01 | 52.78 | 54.34 | 55.27 | 55.86 | 56.06 |
| **22** | 48.45 | 48.67 | 49.33 | 50.35 | 52.03 | 53.89 | 55.73 | 57.38 | 58.35 | 58.98 | 59.20 |
| **23** | 50.87 | 51.12 | 51.88 | 53.02 | 54.89 | 56.91 | 58.86 | 60.58 | 61.58 | 62.23 | 62.44 |
| **24** | 53.95 | 54.20 | 54.92 | 56.04 | 57.91 | 60.00 | 62.08 | 63.95 | 65.08 | 65.80 | 66.05 |
| **25** | 56.61 | 56.89 | 57.72 | 58.97 | 61.03 | 63.25 | 65.41 | 67.31 | 68.42 | 69.14 | 69.38 |
| **26** | 59.50 | 59.78 | 60.61 | 61.85 | 63.89 | 66.10 | 68.24 | 70.12 | 71.22 | 71.93 | 72.17 |
| **27** | 62.47 | 62.74 | 63.56 | 64.82 | 66.90 | 69.20 | 71.48 | 73.51 | 74.72 | 75.50 | 75.76 |
| **28** | 65.24 | 65.50 | 66.29 | 67.50 | 69.53 | 71.80 | 74.07 | 76.12 | 77.35 | 78.15 | 78.41 |
| **29** | 67.62 | 67.90 | 68.75 | 70.04 | 72.17 | 74.50 | 76.80 | 78.83 | 80.04 | 80.81 | 81.07 |
| **30** | 70.12 | 70.40 | 71.23 | 72.51 | 74.64 | 76.99 | 79.33 | 81.42 | 82.67 | 83.48 | 83.75 |
| **31** | 71.98 | 72.29 | 73.19 | 74.55 | 76.79 | 79.21 | 81.57 | 83.65 | 84.87 | 85.66 | 85.92 |
| **32** | 74.45 | 74.74 | 75.58 | 76.87 | 79.01 | 81.38 | 83.74 | 85.85 | 87.11 | 87.92 | 88.19 |
| **33** | 76.40 | 76.71 | 77.61 | 78.98 | 81.21 | 83.62 | 85.96 | 88.00 | 89.20 | 89.97 | 90.23 |
| **34** | 78.00 | 78.31 | 79.24 | 80.65 | 82.97 | 85.48 | 87.92 | 90.07 | 91.33 | 92.14 | 92.41 |
| **35** | 79.79 | 80.09 | 81.00 | 82.38 | 84.64 | 87.12 | 89.56 | 91.71 | 92.98 | 93.80 | 94.07 |
| **36** | 81.68 | 81.97 | 82.84 | 84.17 | 86.37 | 88.78 | 91.16 | 93.26 | 94.51 | 95.32 | 95.59 |
| **37** | 83.14 | 83.41 | 84.24 | 85.51 | 87.64 | 90.01 | 92.39 | 94.52 | 95.80 | 96.63 | 96.91 |
| **38** | 84.15 | 84.42 | 85.25 | 86.52 | 88.64 | 90.99 | 93.35 | 95.46 | 96.73 | 97.55 | 97.82 |
| **39** | 84.93 | 85.22 | 86.09 | 87.41 | 89.60 | 92.00 | 94.38 | 96.48 | 97.73 | 98.54 | 98.81 |
| **40** | 85.22 | 85.52 | 86.40 | 87.75 | 90.00 | 92.50 | 94.99 | 97.23 | 98.56 | 99.43 | 99.72 |

*Table 2 LMS-Generated Fetal Growth Reference (Head Circumference)*

| GA | P_2.5 | P_3 | P_5 | P_10 | P_25 | P_50 | P_75 | P_90 | P_95 | P_97 | P_97.5 |
|---|---|---|---|---|---|---|---|---|---|---|---|
| **15** | 111.64 | 112.17 | 113.66 | 115.77 | 118.89 | 121.91 | 124.57 | 126.73 | 127.94 | 128.69 | 128.94 |
| **16** | 116.28 | 116.75 | 118.20 | 120.50 | 124.56 | 129.43 | 134.71 | 139.85 | 143.13 | 145.34 | 146.10 |
| **17** | 127.09 | 127.62 | 129.22 | 131.76 | 136.20 | 141.44 | 147.04 | 152.40 | 155.77 | 158.03 | 158.80 |
| **18** | 139.73 | 140.36 | 142.25 | 145.15 | 150.01 | 155.43 | 160.87 | 165.77 | 168.71 | 170.62 | 171.26 |
| **19** | 151.20 | 151.93 | 154.07 | 157.30 | 162.49 | 168.00 | 173.26 | 177.81 | 180.45 | 182.14 | 182.70 |
| **20** | 163.79 | 164.41 | 166.27 | 169.10 | 173.81 | 178.98 | 184.09 | 188.64 | 191.34 | 193.09 | 193.67 |
| **21** | 173.33 | 173.99 | 175.99 | 179.04 | 184.12 | 189.73 | 195.29 | 200.27 | 203.23 | 205.14 | 205.78 |
| **22** | 184.59 | 185.25 | 187.22 | 190.24 | 195.29 | 200.89 | 206.48 | 211.50 | 214.50 | 216.45 | 217.10 |
| **23** | 193.96 | 194.67 | 196.79 | 200.05 | 205.47 | 211.48 | 217.47 | 222.84 | 226.04 | 228.12 | 228.81 |
| **24** | 203.69 | 204.46 | 206.74 | 210.25 | 216.08 | 222.55 | 228.98 | 234.74 | 238.17 | 240.40 | 241.14 |
| **25** | 214.16 | 215.02 | 217.56 | 221.39 | 227.63 | 234.33 | 240.81 | 246.46 | 249.76 | 251.88 | 252.59 |
| **26** | 224.92 | 225.73 | 228.15 | 231.85 | 238.04 | 244.89 | 251.71 | 257.83 | 261.47 | 263.84 | 264.63 |
| **27** | 234.91 | 235.80 | 238.43 | 242.43 | 248.96 | 256.00 | 262.84 | 268.83 | 272.34 | 274.59 | 275.34 |
| **28** | 245.42 | 246.23 | 248.64 | 252.36 | 258.58 | 265.50 | 272.43 | 278.67 | 282.40 | 284.82 | 285.64 |
| **29** | 253.17 | 254.05 | 256.67 | 260.66 | 267.26 | 274.49 | 281.61 | 287.92 | 291.65 | 294.07 | 294.87 |
| **30** | 262.21 | 263.07 | 265.62 | 269.53 | 276.04 | 283.23 | 290.38 | 296.76 | 300.57 | 303.03 | 303.86 |
| **31** | 269.11 | 270.02 | 272.73 | 276.86 | 283.68 | 291.14 | 298.49 | 304.99 | 308.84 | 311.33 | 312.16 |
| **32** | 276.82 | 277.69 | 280.26 | 284.23 | 290.88 | 298.29 | 305.72 | 312.43 | 316.44 | 319.06 | 319.93 |
| **33** | 283.73 | 284.58 | 287.13 | 291.06 | 297.64 | 304.98 | 312.34 | 318.99 | 322.97 | 325.56 | 326.43 |
| **34** | 288.23 | 289.16 | 291.94 | 296.20 | 303.26 | 311.04 | 318.74 | 325.60 | 329.68 | 332.32 | 333.21 |
| **35** | 294.43 | 295.31 | 297.95 | 302.01 | 308.81 | 316.37 | 323.93 | 330.74 | 334.81 | 337.45 | 338.34 |
| **36** | 300.33 | 301.20 | 303.78 | 307.74 | 314.36 | 321.71 | 329.04 | 335.62 | 339.55 | 342.11 | 342.96 |
| **37** | 305.03 | 305.87 | 308.39 | 312.28 | 318.78 | 326.03 | 333.29 | 339.84 | 343.77 | 346.32 | 347.17 |
| **38** | 308.02 | 308.84 | 311.27 | 315.03 | 321.39 | 328.54 | 335.79 | 342.39 | 346.37 | 348.97 | 349.85 |
| **39** | 308.71 | 309.69 | 312.60 | 316.97 | 324.04 | 331.58 | 338.81 | 345.08 | 348.73 | 351.06 | 351.83 |
| **40** | 310.40 | 311.34 | 314.17 | 318.50 | 325.69 | 333.61 | 341.45 | 348.44 | 352.59 | 355.28 | 356.18 |

*Table 3 LMS-Generated Fetal Growth Reference (Abdominal Circumference)*

| GA | P_2.5 | P_3 | P_5 | P_10 | P_25 | P_50 | P_75 | P_90 | P_95 | P_97 | P_97.5 |
|---|---|---|---|---|---|---|---|---|---|---|---|
| **15** | 88.64 | 89.45 | 91.70 | 94.77 | 99.11 | 103.15 | 106.61 | 109.37 | 110.88 | 111.83 | 112.13 |
| **16** | 95.84 | 96.32 | 97.76 | 100.04 | 104.02 | 108.71 | 113.70 | 118.46 | 121.45 | 123.45 | 124.13 |
| **17** | 105.72 | 106.25 | 107.85 | 110.38 | 114.80 | 119.99 | 125.50 | 130.75 | 134.04 | 136.23 | 136.98 |
| **18** | 113.46 | 114.33 | 116.89 | 120.69 | 126.72 | 133.02 | 138.95 | 144.03 | 146.95 | 148.81 | 149.43 |
| **19** | 125.80 | 126.67 | 129.20 | 132.94 | 138.87 | 145.02 | 150.80 | 155.71 | 158.53 | 160.33 | 160.92 |
| **20** | 139.87 | 140.52 | 142.47 | 145.50 | 150.66 | 156.53 | 162.55 | 168.08 | 171.44 | 173.65 | 174.39 |
| **21** | 148.79 | 149.55 | 151.79 | 155.21 | 160.86 | 167.06 | 173.18 | 178.61 | 181.83 | 183.91 | 184.60 |
| **22** | 158.96 | 159.75 | 162.08 | 165.63 | 171.47 | 177.83 | 184.06 | 189.55 | 192.79 | 194.88 | 195.58 |
| **23** | 168.33 | 169.13 | 171.54 | 175.23 | 181.35 | 188.08 | 194.74 | 200.67 | 204.19 | 206.47 | 207.23 |
| **24** | 177.34 | 178.18 | 180.70 | 184.57 | 191.04 | 198.21 | 205.38 | 211.82 | 215.67 | 218.17 | 219.01 |
| **25** | 185.75 | 186.79 | 189.86 | 194.44 | 201.80 | 209.59 | 217.01 | 223.41 | 227.13 | 229.50 | 230.28 |
| **26** | 195.57 | 196.60 | 199.66 | 204.27 | 211.69 | 219.60 | 227.18 | 233.75 | 237.57 | 240.02 | 240.83 |
| **27** | 205.94 | 207.02 | 210.23 | 215.09 | 223.03 | 231.60 | 239.93 | 247.23 | 251.51 | 254.27 | 255.18 |
| **28** | 217.06 | 218.09 | 221.18 | 225.90 | 233.71 | 242.30 | 250.77 | 258.31 | 262.78 | 265.67 | 266.63 |
| **29** | 224.02 | 225.19 | 228.67 | 233.95 | 242.65 | 252.11 | 261.37 | 269.55 | 274.37 | 277.48 | 278.52 |
| **30** | 233.79 | 235.02 | 238.66 | 244.19 | 253.25 | 263.08 | 272.67 | 281.10 | 286.07 | 289.26 | 290.33 |
| **31** | 242.58 | 243.92 | 247.85 | 253.75 | 263.29 | 273.44 | 283.16 | 291.59 | 296.49 | 299.63 | 300.67 |
| **32** | 252.68 | 254.04 | 258.03 | 264.04 | 273.77 | 284.14 | 294.11 | 302.75 | 307.79 | 311.02 | 312.09 |
| **33** | 261.94 | 263.22 | 267.02 | 272.84 | 282.51 | 293.18 | 303.75 | 313.18 | 318.78 | 322.42 | 323.63 |
| **34** | 268.17 | 269.59 | 273.81 | 280.21 | 290.72 | 302.11 | 313.23 | 323.02 | 328.78 | 332.48 | 333.72 |
| **35** | 278.50 | 279.87 | 283.94 | 290.20 | 300.61 | 312.13 | 323.60 | 333.86 | 339.97 | 343.94 | 345.27 |
| **36** | 288.37 | 289.65 | 293.49 | 299.43 | 309.45 | 320.73 | 332.15 | 342.53 | 348.79 | 352.89 | 354.26 |
| **37** | 296.03 | 297.27 | 301.00 | 306.78 | 316.60 | 327.75 | 339.12 | 349.56 | 355.89 | 360.04 | 361.44 |
| **38** | 300.85 | 302.15 | 306.04 | 312.07 | 322.23 | 333.67 | 345.26 | 355.80 | 362.15 | 366.31 | 367.70 |
| **39** | 306.14 | 307.43 | 311.29 | 317.26 | 327.28 | 338.51 | 349.82 | 360.06 | 366.21 | 370.22 | 371.57 |
| **40** | 305.72 | 307.24 | 311.78 | 318.74 | 330.33 | 343.15 | 355.89 | 367.30 | 374.10 | 378.51 | 379.98 |

*Table 4 LMS-Generated Fetal Growth Reference (Femur Length)*

| GA | P_2.5 | P_3 | P_5 | P_10 | P_25 | P_50 | P_75 | P_90 | P_95 | P_97 | P_97.5 |
|---|---|---|---|---|---|---|---|---|---|---|---|
| **15** | 15.38 | 15.45 | 15.68 | 16.03 | 16.67 | 17.45 | 18.31 | 19.17 | 19.72 | 20.10 | 20.23 |
| **16** | 17.13 | 17.25 | 17.64 | 18.26 | 19.36 | 20.69 | 22.16 | 23.61 | 24.53 | 25.16 | 25.38 |
| **17** | 19.49 | 19.66 | 20.20 | 21.02 | 22.43 | 24.01 | 25.62 | 27.09 | 27.97 | 28.56 | 28.75 |
| **18** | 22.34 | 22.54 | 23.11 | 23.99 | 25.46 | 27.08 | 28.70 | 30.15 | 31.02 | 31.58 | 31.77 |
| **19** | 25.79 | 25.99 | 26.55 | 27.41 | 28.78 | 30.23 | 31.61 | 32.81 | 33.50 | 33.95 | 34.10 |
| **20** | 28.81 | 28.99 | 29.52 | 30.33 | 31.68 | 33.17 | 34.64 | 35.96 | 36.74 | 37.25 | 37.41 |
| **21** | 31.53 | 31.70 | 32.21 | 33.01 | 34.34 | 35.83 | 37.33 | 38.69 | 39.51 | 40.04 | 40.22 |
| **22** | 33.79 | 33.98 | 34.57 | 35.45 | 36.90 | 38.47 | 40.01 | 41.36 | 42.16 | 42.68 | 42.85 |
| **23** | 36.16 | 36.36 | 36.96 | 37.87 | 39.38 | 41.02 | 42.64 | 44.07 | 44.92 | 45.46 | 45.64 |
| **24** | 38.28 | 38.50 | 39.17 | 40.17 | 41.78 | 43.49 | 45.11 | 46.51 | 47.33 | 47.85 | 48.02 |
| **25** | 40.56 | 40.79 | 41.47 | 42.49 | 44.14 | 45.91 | 47.61 | 49.10 | 49.96 | 50.52 | 50.70 |
| **26** | 42.62 | 42.87 | 43.60 | 44.69 | 46.44 | 48.30 | 50.07 | 51.60 | 52.49 | 53.06 | 53.25 |
| **27** | 45.29 | 45.53 | 46.24 | 47.31 | 49.04 | 50.90 | 52.69 | 54.25 | 55.17 | 55.75 | 55.95 |
| **28** | 47.52 | 47.77 | 48.50 | 49.58 | 51.34 | 53.20 | 54.99 | 56.53 | 57.42 | 57.99 | 58.19 |
| **29** | 49.10 | 49.39 | 50.22 | 51.45 | 53.39 | 55.39 | 57.26 | 58.84 | 59.75 | 60.33 | 60.52 |
| **30** | 51.94 | 52.19 | 52.91 | 54.00 | 55.79 | 57.72 | 59.60 | 61.24 | 62.21 | 62.83 | 63.04 |
| **31** | 53.21 | 53.49 | 54.34 | 55.60 | 57.59 | 59.67 | 61.61 | 63.27 | 64.22 | 64.83 | 65.03 |
| **32** | 55.87 | 56.13 | 56.89 | 58.04 | 59.89 | 61.85 | 63.74 | 65.37 | 66.32 | 66.92 | 67.13 |
| **33** | 57.54 | 57.81 | 58.61 | 59.80 | 61.71 | 63.73 | 65.65 | 67.30 | 68.25 | 68.86 | 69.07 |
| **34** | 59.05 | 59.33 | 60.15 | 61.37 | 63.33 | 65.38 | 67.32 | 68.98 | 69.94 | 70.56 | 70.76 |
| **35** | 61.01 | 61.28 | 62.07 | 63.26 | 65.18 | 67.22 | 69.17 | 70.86 | 71.83 | 72.46 | 72.67 |
| **36** | 62.74 | 63.01 | 63.81 | 64.99 | 66.88 | 68.86 | 70.74 | 72.35 | 73.29 | 73.88 | 74.08 |
| **37** | 64.35 | 64.60 | 65.34 | 66.46 | 68.28 | 70.26 | 72.17 | 73.85 | 74.84 | 75.47 | 75.68 |
| **38** | 65.54 | 65.79 | 66.54 | 67.68 | 69.53 | 71.53 | 73.47 | 75.16 | 76.16 | 76.80 | 77.01 |
| **39** | 66.81 | 67.05 | 67.79 | 68.90 | 70.72 | 72.69 | 74.60 | 76.27 | 77.26 | 77.89 | 78.10 |
| **40** | 67.50 | 67.75 | 68.49 | 69.61 | 71.44 | 73.40 | 75.31 | 76.98 | 77.96 | 78.59 | 78.80 |

*Table 5 LMS-Generated Fetal Growth Reference (Estimated Fetal Weight)*

| GA | P_2.5 | P_3 | P_5 | P_10 | P_25 | P_50 | P_75 | P_90 | P_95 | P_97 | P_97.5 |
|---|---|---|---|---|---|---|---|---|---|---|---|
| **15** | 108.2 | 109.1 | 111.7 | 116.0 | 124.1 | 134.5 | 146.9 | 160.2 | 169.4 | 176.0 | 178.4 |
| **16** | 128.5 | 129.5 | 132.8 | 138.1 | 147.7 | 159.9 | 174.0 | 188.5 | 198.3 | 205.1 | 207.5 |
| **17** | 149.2 | 151.0 | 156.6 | 165.5 | 180.9 | 199.2 | 218.5 | 236.9 | 248.3 | 255.9 | 258.5 |
| **18** | 184.9 | 187.4 | 194.8 | 206.3 | 225.7 | 247.7 | 270.0 | 290.4 | 302.7 | 310.8 | 313.5 |
| **19** | 226.0 | 229.1 | 238.5 | 252.6 | 275.7 | 300.4 | 324.4 | 345.5 | 357.8 | 365.8 | 368.4 |
| **20** | 285.9 | 288.7 | 297.2 | 310.7 | 334.4 | 362.5 | 392.5 | 421.2 | 439.1 | 451.1 | 455.2 |
| **21** | 338.6 | 342.1 | 352.6 | 369.1 | 397.9 | 431.5 | 466.9 | 500.3 | 520.9 | 534.7 | 539.3 |
| **22** | 397.2 | 401.5 | 414.6 | 434.9 | 469.7 | 509.5 | 550.5 | 588.4 | 611.4 | 626.6 | 631.7 |
| **23** | 460.9 | 466.0 | 481.5 | 505.7 | 547.3 | 595.4 | 645.1 | 691.4 | 719.7 | 738.4 | 744.7 |
| **24** | 531.1 | 537.3 | 556.0 | 585.2 | 635.3 | 692.8 | 752.2 | 807.2 | 840.8 | 862.9 | 870.3 |
| **25** | 608.0 | 616.1 | 640.2 | 677.3 | 739.6 | 809.1 | 878.9 | 941.9 | 979.6 | 1004.2 | 1012.4 |
| **26** | 704.1 | 713.0 | 739.9 | 781.6 | 852.2 | 932.0 | 1012.9 | 1086.7 | 1131.2 | 1160.3 | 1170.1 |
| **27** | 817.0 | 827.3 | 858.5 | 906.9 | 989.5 | 1083.5 | 1179.7 | 1268.0 | 1321.5 | 1356.7 | 1368.5 |
| **28** | 947.4 | 958.6 | 992.5 | 1045.3 | 1135.7 | 1239.1 | 1345.4 | 1443.5 | 1503.2 | 1542.5 | 1555.7 |
| **29** | 1039.4 | 1053.5 | 1096.0 | 1161.8 | 1272.8 | 1397.8 | 1524.2 | 1639.1 | 1708.3 | 1753.4 | 1768.6 |
| **30** | 1194.8 | 1209.7 | 1254.7 | 1325.0 | 1445.7 | 1584.5 | 1728.1 | 1861.2 | 1942.5 | 1996.1 | 2014.1 |
| **31** | 1315.0 | 1333.0 | 1386.9 | 1469.9 | 1609.2 | 1765.0 | 1921.5 | 2062.8 | 2147.5 | 2202.7 | 2221.2 |
| **32** | 1492.4 | 1511.0 | 1566.8 | 1653.3 | 1799.9 | 1965.6 | 2133.9 | 2287.4 | 2380.1 | 2440.8 | 2461.1 |
| **33** | 1646.3 | 1666.3 | 1726.3 | 1819.9 | 1979.9 | 2162.9 | 2350.9 | 2524.2 | 2629.7 | 2699.1 | 2722.4 |
| **34** | 1773.5 | 1796.6 | 1865.9 | 1973.5 | 2156.0 | 2362.4 | 2572.4 | 2764.1 | 2880.0 | 2955.8 | 2981.3 |
| **35** | 1966.9 | 1990.5 | 2061.6 | 2172.5 | 2362.3 | 2579.7 | 2803.4 | 3009.9 | 3135.7 | 3218.5 | 3246.4 |
| **36** | 2173.4 | 2196.6 | 2266.9 | 2377.2 | 2567.9 | 2788.9 | 3019.5 | 3235.0 | 3367.6 | 3455.3 | 3485.0 |
| **37** | 2343.7 | 2366.9 | 2437.0 | 2547.7 | 2740.2 | 2965.7 | 3203.5 | 3428.0 | 3567.3 | 3659.9 | 3691.3 |
| **38** | 2465.5 | 2489.9 | 2563.9 | 2680.3 | 2882.4 | 3118.3 | 3366.0 | 3599.1 | 3743.3 | 3839.0 | 3871.4 |
| **39** | 2587.6 | 2614.0 | 2693.3 | 2816.9 | 3028.2 | 3269.6 | 3517.9 | 3746.8 | 3886.2 | 3977.8 | 4008.7 |
| **40** | 2605.4 | 2634.1 | 2720.8 | 2856.7 | 3090.9 | 3361.6 | 3643.1 | 3905.3 | 4066.2 | 4172.4 | 4208.3 |
